# Fabrication of Super-Hydrophobic Microchannels via Strain-Recovery Deformations of Polystyrene and Oxygen Reactive Ion Etch

**DOI:** 10.3390/ma6083610

**Published:** 2013-08-19

**Authors:** Anirban Chakraborty, Mingming Xiang, Cheng Luo

**Affiliations:** Department of Mechanical and Aerospace Engineering, University of Texas, Arlington, TX 76019, USA; E-Mails: anirban.chakraborty@gmail.com (A.C.); mingming.xiang@mavs.uta.edu (M.X.)

**Keywords:** shape-memory polymer, super-hydrophobic microchannels, hybrid micro/nanostructures

## Abstract

In this article, we report a simple approach to generate micropillars (whose top portions are covered by sub-micron wrinkles) on the inner surfaces of polystyrene (PS) microchannels, as well as on the top surface of the PS substrate, based on strain-recovery deformations of the PS and oxygen reactive ion etch (ORIE). Using this approach, two types of micropillar-covered microchannels are fabricated. Their widths range from 118 μm to 132 μm, depths vary from 40 μm to 44 μm, and the inclined angles of their sidewalls are from 53° to 64°. The micropillars enable these microchannels to have super-hydrophobic properties. The contact angles observed on the channel-structured surfaces are above 162°, and the tilt angles to make water drops roll off from these channel-structured substrates can be as small as 1°.

## 1. Introduction

It is observed that the leaf surfaces of some lotuses have dual-size roughnesses, *i.e.*, micropillars are covered by sub-micron pillars [1,2]. These hierarchical structures make a leaf surface have super-hydrophobic properties, *i.e.*, high contact angle and low contact angle hysteresis. The high contact angle makes water, for example, have a form of drops on a surface, and low contact angle hysteresis enables a water drop to easily roll off from this surface, taking away dusts. Accordingly, the lotus exhibits a self-cleaning effect.

As a water drop is placed on a micro/nanostructure-formed surface, the wetting may be either in a Wenzel [3] or Cassie-Baxter state [4]. In the Wenzel state, the drop completely penetrates between the surface structures, while in the Cassie-Baxter state, air is trapped between these structures and the drop stays on top of the surfaces and trapped air. Due to the pinning effect, a water drop is difficult to get off from a surface when the wetting is in the Wenzel state. In contrast, the Cassie-Baxter state reduces solid/liquid contact areas, and thus enables a water drop to easily roll off from a hydrophobic surface [1,2], and also reduces drag friction of a liquid flow on this surface [5,6,7]. The wetting is normally in the Cassie-Baxter state in the case of lotus [1,2]. In the Cassie-Baxter state [4], we have $\text{cos}\theta =f_{SA}(\text{cos}\theta_{0}+1)-1$, where *θ* and $\theta_{0}$ are the contact angles for rough and flat surfaces, respectively, and $f_{SA}$ corresponds to the ratio of the top surface of the roughness in contact with the liquid with the apparent surface of the substrate. It is observed from this equation that, given that $\theta_{0}$ is greater than 90° (*i.e*., the surface coating is hydrophobic), *θ* increases with the decrease in $f_{SA}$. Also, it has been demonstrated in [8] that the contact angle hysteresis also decreases with the decreasing $f_{SA}$. These results analytically justify why the hybrid micro/nanostructures on a lotus surface make a water drop easily move away from this surface. Furthermore, according to [5,6,7], drag friction to a liquid flow also decreases with the decrease in $f_{SA}$.

Motivated by surface structures on a lotus, in the past two decades researchers have constructed artificial surfaces with super-hydrophobic properties by creating hybrid micro/nanostructures on different surfaces [8,9,10,11,12,13,14]. On the other hand, their focus is on the generation of dual-size roughnesses on flat surfaces, while little work has been done to create such structures on the non-flat inner surfaces of microchannels.

Existing photolithographic approaches, such as ultra-violet (UV), electron-beam, X-ray, and ion-beam, employ vertical light exposure to transfer patterns. They are suited at fabricating micro or nanostructures on flat substrates, but not capable of patterning the sidewalls of substrate structures. Meanwhile, unconventional approaches, such as inclined UV lithography [15,16,17], shadow-masking [18,19,20], chemical synthesis [21] and modified hot-embossing approaches [22,23], are limited to create only micro or nanopatterns on the sidewalls of a microstructure, and it is difficult to apply them to fabricate hybrid micro/nanostructures on curved surfaces. In a previous work [24], using strain-recovery properties of a PS sheet and oxygen reactive ion etch (ORIE), we generated Ag dots and lines on the inner surfaces of microchannels. The surfaces of these dots and lines were covered with sub-micron wrinkles. Since the Ag dots and lines are thin (their thicknesses are less than 1 μm), water is easy to have contact with the bottoms of the grooves located between these patterns, making the micropatterns losing their function of reducing $f_{SA}$. To avoid this contact problem for further decreasing $f_{SA}$, in this work, we modify the fabrication approach of our previous work [24], and make it suited at generating micropillars on the inner surfaces of a microchannel. As in the previous work, the top surfaces of these micropillars are still structured with much smaller wrinkles, forming hybrid micro/nanostructures on the inner surfaces of microchannels. On the other hand, since the micropillars are much taller than microdots and microlines, water just suspends between micropillars, and does not have direct contact with the base of the grooves. Accordingly, in addition to wrinkles, micropillars also contribute to the reduction of $f_{SA}$.

Shape Memory Polymers (SMP) are materials which are able to recover their original shapes upon application of a stimulus such as temperature (cooling/heating), light and magnetic field [25]. The PS material used is a thermal SMP [24,26,27,28,29], and has capability to recover from its deformed shape to original shape upon heating to above its glass-transition temperature (*T_g_*). The PS sheet tested in this work is a commercial product used for packaging applications (Multi Plastics Incorporate). It has been processed into the present form in its manufacturing site by heating a PS sheet to a temperature a few degrees below *T_g_* and stretching the sheet into a 25-µm-thick film along two perpendicular directions [26]. The degree of shrinking approximately equals the draw ratio of the orientation process. In this work, the PS sheet was used as received. Its *T_g_* and melting temperature were 95 and 270 °C, respectively. The recovery temperature used was 130 °C. According to our tests on mm-scale rectangular blocks, which were cut off from the PS sheets, after recovery a pre-stretched PS block reduced its two lateral dimensions by factors of 4.4 and 4.0, respectively, implying that the pre-strains along the lateral directions were in the range of 4.0 to 4.4. The recovered PS block increased the height by a factor of 20.7.

The outline of this note is as follows. Fabrication procedure is introduced in Section 2. Experimental results are presented and discussed in Section 3. Finally, this work is summarized in Section 4.

## 2. Fabrication Procedure

**Figure 1:**
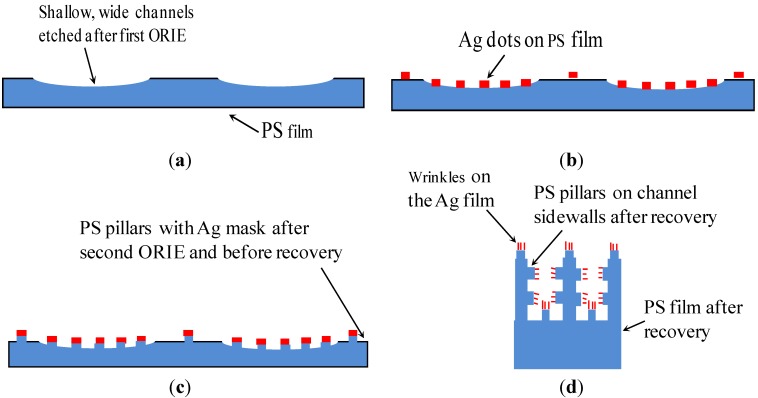
Schematics of the four-step procedure used to fabricate super-hydrophobic microchannels. (**a**) Generate shallow, wide channels; (**b**) Create Ag microdots on the substrate; (**c**) Produce micropillars; and (**d**) Complete the fabrication through strain recovery of the PS substrate (not to scale).

As shown in Figure 1, the fabrication procedure used in the new approach includes four basic steps. In our previous work [24], the first, second and fourth steps were used to generate Ag micropatterns on the inner surfaces of a channel. In this work, the third step is added in the fabrication procedure to produce micropillars on the finally generated channels.

In the first step (Figure 1a), a mm-scale block was cut off from a PS sheet and placed on a Teflon-coated glass slide. The glass slide was 75 mm long, 25 mm wide and 1 mm thick (Fisher Scientific Company, Waltham, MA, USA). Subsequently, shallow channels were generated on a mm-scale PS block using ORIE as follows. A stencil with hollow channels was placed on the top surface of the PS block. Subsequently, part of the PS located underneath the hollow channel was partially etched by the ORIE using the stencil as a mask. Subsequently, the stencil was removed and an array of shallow channels was left on the top surface of the PS block. The ORIE was conducted in a commercial machine (Model: Plasma Lab RIE, Plasma Technology Inc., Torrance, CA, USA). Pressure was maintained at 170 m Torr, oxygen flow rate was fixed at 10 standard cubic centimeters per minute, and the RF power ranged from 140 to 180 W. The resulting etch rate of the PS was around 0.02 μm min^−1^. The ORIE technique has been previously applied by us to generate similar channels in a PS film [24], as well as to remove undesired PS films [30].

In the second step (Figure 1b), Ag microdots were deposited on the mm-scale PS block as follows: (i) a stencil with through-holes was placed on top of the PS block; (ii) a Ag film, whose thickness was in the range of 500 nm to 600 nm**,** was coated on the block by sputter deposition; and (iii) after sputtering, the stencil was removed and Ag microdots were left on the top surface of the PS substrate.

In the third step (Figure 1c), using Ag microdots as masking patterns, micropillars were generated on the PS block using oxygen reactive ion etch (RIE). The PS film that were not covered by the Ag microdots were etched to a depth of about 2 μm, while the portion of the PS film underneath the Ag microdots were not etched. Accordingly, PS micropillars of height 2 μm were formed on the PS film, and they were capped by the Ag microdots.

In the fourth step (Figure 1d), the heights and widths of the PS micropillars were increased and decreased, respectively. The grooves between the PS micropillars also experienced the same changes in the dimensions as the PS micropillars. All these changes were caused by the strain recovery of the pre-stretched PS film at 130 °C. As such, the desired dual-size roughnesses were created on the PS surfaces.

## 3. Experimental and Discussions

### 3.1. Preliminary Tests

**Figure 2:**
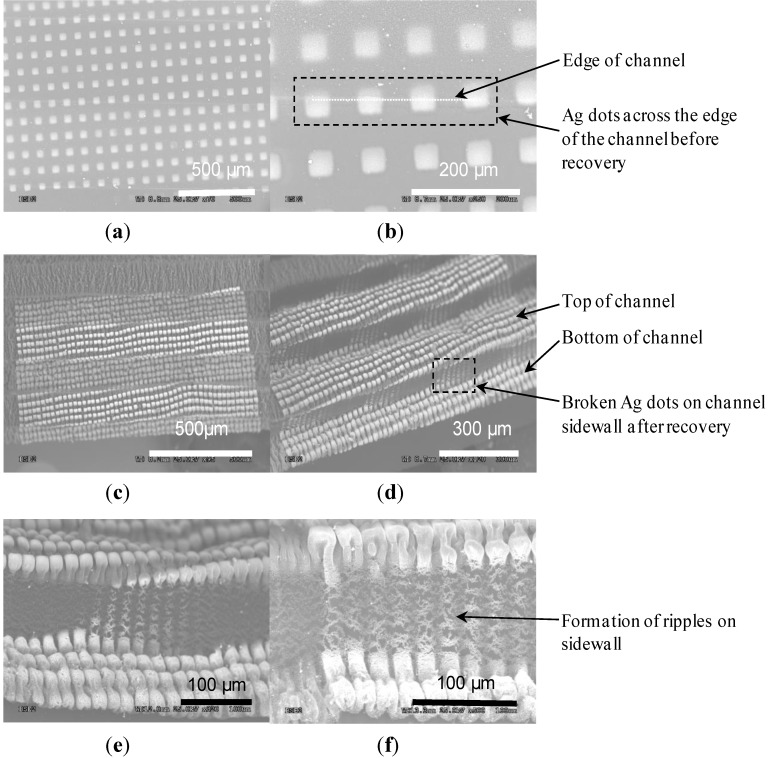
Scanning electron microscopy (SEM) images of 500-µm-wide channels with 50 × 50 µm^2^ Ag dots. (**a**,**b**) Top view of Ag dots before recovery; (**c**,**d**) Top and side view of the sample after recovery; (**e**,**f**) Close-up of the vertical sidewall with the broken Ag dots and ripples.

**Figure 3:**
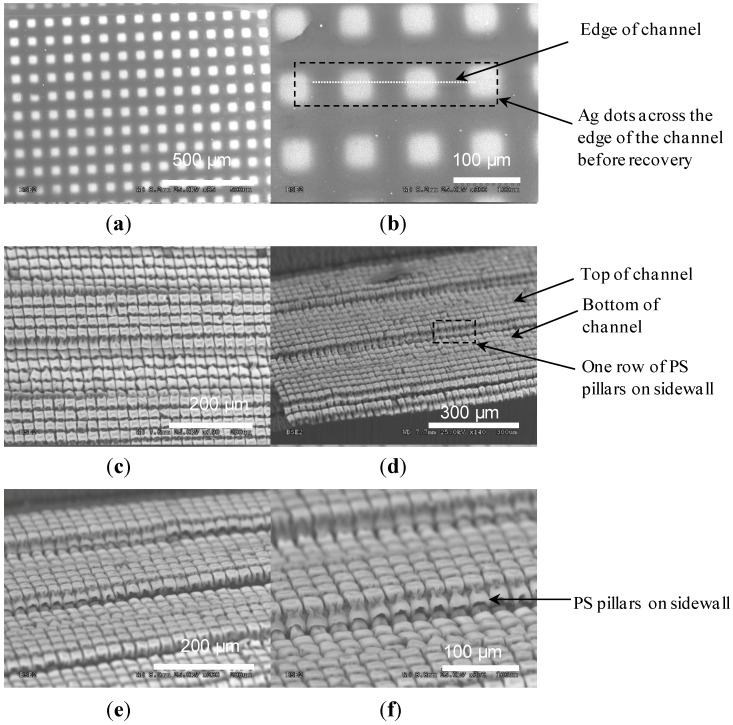
SEM images of 500-µm-wide channels with 50 × 50 µm^2^ Ag dots. (**a**,**b**) Top view of Ag dots before recovery; (**c**,**d**) Top and side views of the sample after recovery; (**e**,**f**) Close-up views of the vertical sidewall with one row of polystyrene (PS) pillars.

Figure 2 gives preliminary results generated using the aforementioned fabrication procedure. The initial depth of the channel was 4–5 µm and, after strain recovery, it was 80–100 µm. The sidewall angles before and after recovery were 16° and 85°, respectively. Before recovery, a row of 50 × 50 µm2 Ag dots with the spacing of 50 µm ran across an edge of the channel (Figure 2a,b). During strain recovery, the channel sidewall was stretched, causing this row of Ag dots to break across the sidewall and also resulting in the formation of ripples along the channel sidewall (Figure 2c–f). Both defects were solved by reducing the etch depths of the channels to 2–3 µm using a shorter ORIE duration, which decreased the corresponding strain and stress that the Ag dots suffered during the recovery process (Figure 3). After recovery, the depths of the channels were reduced to 25–30 µm with an inclination degree of 87°, and micropillars were obtained both on the top surface of the substrate and inner surfaces of channels. Cracking of the Ag dots and formation of ripples were not observed in this case. On the other hand, it was noticed that the spacings between recovered micropillars were small, which were less than 5 µm (Figure 3c–f). To enlarge such spacings for larger reduction in the value of $f_{SA}$, initial spacings should be increased. Thus, in the follow-up tests, the initial spacings were increased from 50 to 110 µm or above.

### 3.2. Generated Microchannels

**Figure 4:**
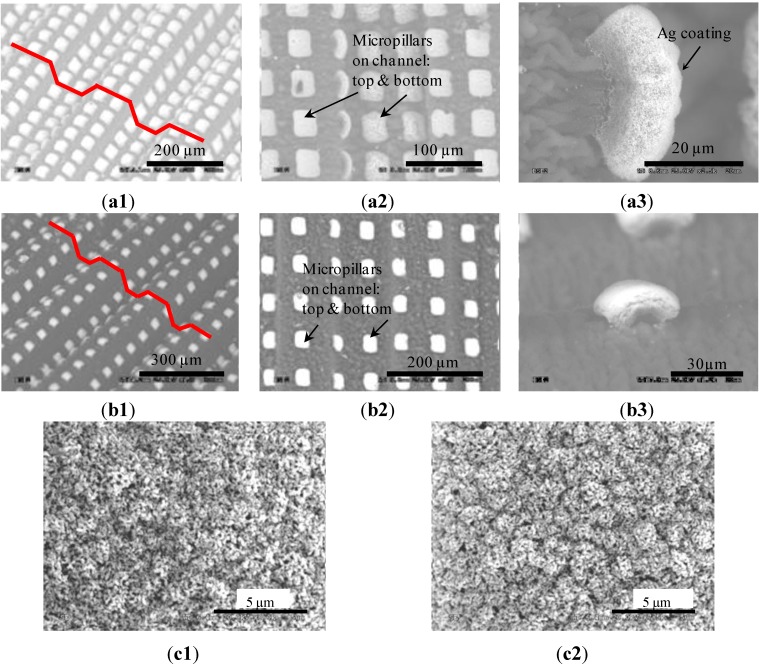
Two arrays of generated channels. (**a1**) 3-D; (**a2**) Top; and (**a3**) Close-up views of Channels I; (**b1**) 3-D; (**b2**) Top; and (**b3**) Close-up views of Channels II; Sub-micron wrinkles generated on the tops of Pillars. (**c1**) I; and (**c2**) II. (All are SEM images).

Based on the understanding gained in the preliminary tests, two arrays of microchannels were fabricated on different PS sheets, and, for simplicity, are called Channels I and II according to their order shown in Figure 4. Each array included 10 channels. Before strain recovery, all the channels were about 540 µm wide, 2.5 µm deep and 10 mm long, and micropillars had the same dimensions of 100 × 100 µm^2^. On the other hand, the micropillars on Channels I and II had spacings of 100 and 200 µm, respectively, and are referred to as Pillars I and II thereafter. After recovery, the two arrays of channels had the same lengths of 3 mm but differed in their widths and depths. Channels I and II were, respectively, 40 and 44 µm deep and their openings were 118 and 132 µm wide. This implies that, during recovery, the widths of the microchannels were reduced by a factor of 3.7–4.2 and heights increased by a factor of 16.0–17.6. These values are close to their counterparts in the recovery case of an as-received PS film, which has a lateral shrinkage ratio of 4–4.4 and a vertical increase factor of 20.7. The slight difference may be caused by the Ag dots located on the top of the micropillars. These dots attempted to reduce the recovery deformations of the PS film. Let *θ_i_* and *θ_f_* denote the inclined angles of a channel before and after strain recovery, respectively. The two ar`rays of the channels had the same values of *θ_i_*, which was around 1°. After recovery, the measured values of *θ_f_* were 53° and 64°, respectively. In a previous work [24], we also derived a theoretical relation between *θ_i_* and *θ_f_* as given below:

(1) $$\theta_{f}={\text{tan}}^{-1}[(\frac{y}{x})\text{tan}\theta_{i}]$$

where *y* and *x* denote the elongation ratios along the vertical and horizontal directions, separately. According to this relation, the predicted values of *θ_f_* for both channels are 52°, close to the experimentally measured ones. Equation (1) was originally derived for the case that the channel sidewalls do not have any patterns [24]. In this work, the channel sidewalls have micropillars and also suffer an additional ORIE process, which should affect the accuracy of Equation (1). The consideration of these factors should produce a better model, which we leave to a future investigation. On the other hand, according to the above comparison, this relation could still be applied to estimate the value of *θ_f_* in the current case.

### 3.3. Recovered Micropillars

After recovery, Pillars I and II were formed on the inner surfaces of the corresponding channels, as well as on the top surfaces of the substrates (Figure 4). The pillar heights were increased to 18.2 and 19.3 µm, respectively, and lateral dimensions became 28.5 × 37.1 µm^2^ and 29.2 × 37.7 µm^2^. These results imply that the heights of the recovered micropillars were increased by a factor of 9.1–9.7, and that their lateral dimensions were reduced by a factor of 2.6 to 3.5. These values are much lower than their counterparts in the recovery case of an as-received PS film. As discussed below, this difference is mainly induced by the Ag dots located on the top of these micropillars.

As observed from Figure 4a3,b3, a recovered micropillar has the shape of a mushroom, and the top portion of the micropillar was wrapped inside the Ag coating. At the recovery temperature, which was above *T_g_* of PS, the PS was softened, while the Ag remained solid and was rigid. Consequently, the recovery stress of the PS was large enough to deform the softened PS but had less effect on the Ag patterns. According to [31], the recovery stresses of SMPs were normally between 0.9 and 2.9 MPa (10 kgf cm^−2^ and 30 kgf cm^−2^). However, Young’s modulus of Ag is around 83.0 GPa. Consequently, the recovery did not have a strong impact on the shapes and dimensions of the Ag patterns. On the other hand, such a stress was large enough to trigger the strain recovery of the PS. Accordingly, during strain recovery, the lateral dimensions of a PS micropillar were still reduced, and the height was increased. Since the Ag dot moved together with the underlying PS, its opposite edges were brought towards each other by the recovered PS while its center was pushed up by the underneath PS, forming a curved shape.

Wrinkles could arise in metal skins when their soft substrates had large shrinking deformations [24,28,32,33]. Accordingly, biaxial wrinkles were generated on the top of the two types of micropillars (Figure 4). The wavelengths of the wrinkles were 0.2–2.7 µm (Figure 4c1) and 0.1–1.8 µm (Figure 4c2) for Pillars I and II, respectively. Since the wavelengths are small, the wrinkles look like densely-distributed particles (Figure 4c). The critical conditions for biaxial wrinkling may be estimated as [32]:

(2) $$\lambda_{c}=2\pi h{\left[\frac{4E_{m}(1-{\nu_{p}}^{2})}{3E_{p}(1-{\nu_{m}}^{2})}\right]}^{1/3}$$

(3) $$\sigma_{c}=\frac{1}{4}{\left(\frac{4E_{m}}{1-{\nu_{m}}^{2}}\right)}^{1/3}{\left(\frac{3E_{p}}{1-{\nu_{p}}^{2}}\right)}^{2/3}$$

(4) $$\varepsilon_{c}={\left[\frac{3E_{p}(1-{\nu_{m}}^{2})}{4E_{m}(1-{\nu_{p}}^{2})}\right]}^{2/3}$$

where *λ_c_* is the critical wavelength when the wrinkles begin to appear; *σ_c_* is the critical compressive stress; *ε_c_* is the critical compressive strain in the metal film; and *h* is the thickness of the metal film; *E_m_* and *E_p_* represent Young’s modules of the metal film and its soft substrate, respectively. *ν_m_* and *ν_p_* are Poisson’s ratios of the metal film and its soft substrate. For Ag, *E_m_* and *ν_m_* are 83 GPa and 0.37, respectively. Their values for PS are 3.5 GPa and 0.45. By Equation (2), *λ_c_* = 0.9 µm. The measured wavelengths are in the same order as this value. According to Equations (3) and (4), we have *σ_c_* = 7.9 GPa and *ε_c_* = 10%. These values give a sense of the stresses and strains that the Ag patterns have experienced during the recovery processes.

### 3.4. Wetting Tests

We explored the wetting situations on the channel-structured surfaces. For the purpose of comparison, we also considered those on as-received PS sheets before and after the sheets were recovered. Since both Ag and PS are hydrophilic materials, all the substrates tested in the tests were spin-coated with Teflon which is a highly water-repellent material [34]. In order to preserve the surface morphologies of the recovered microchannel surfaces, the coated Teflon layers are less than 10 nm thick. Water drops used in the tests have volumes 5 to 7 µL. The contact angles are determined using MB-Ruler software of Dance Patterns Company (Iffezheim, Germany) with an error of 2°.

**Figure 5:**
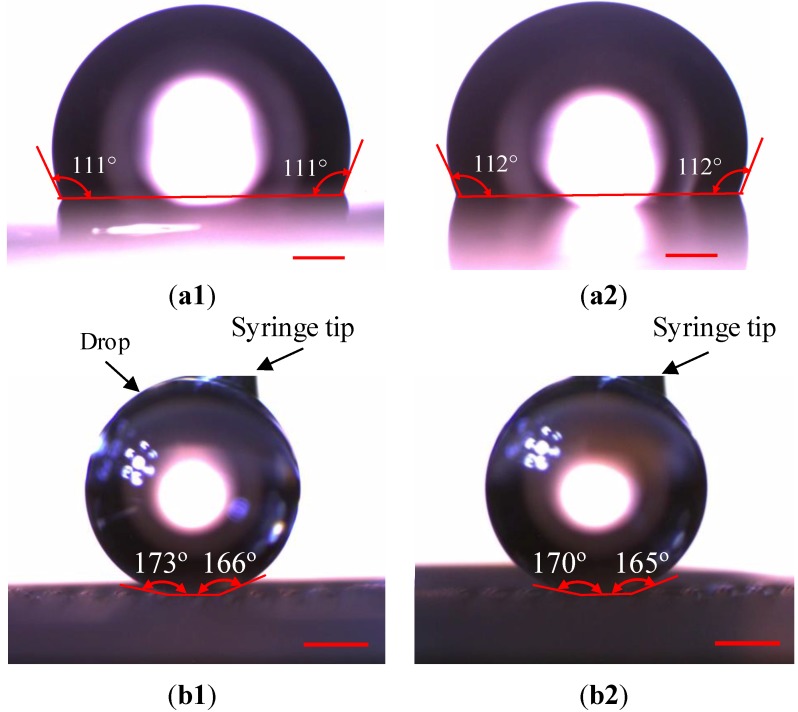
Contact angles on as-received PS sheets. (**a**) Before; and (**b**) After recovery; as well as those on surfaces structured with Channels (**b1**) I and (**b2**) II. The scale bars represent 450 µm.

We first examined the wetting situations on as-received PS sheets. As observed from Figure 5a, the contact angles before and after recovery of the PS sheets were 111° and 112°, respectively, which are about the same. Since contact angles are related to surface roughness, this result indicates that, when the PS sheet does not suffer any surface treatment, the strain recovery has little influence on the surface roughness. The advancing and receding angles measured on an as-received PS sheet were 116° and 97°, respectively, before its recovery. After recovery, the advancing and receding angles became 117° and 97°. The contact angle hystereses were 19° and 20°, separately, in these two cases.

We then measured the contact angles on Channels I and II surfaces. Since water drops were easy to get off from these surfaces, a drop was fixed on a surface using a syringe to make the corresponding measurement. All the measured contact angles are above 164° (Figure 5b), much higher than those on as-received PS sheets. Since the recovery deformations are not ideally uniform, the generated micropillars or channels may not have the same dimensions across the corresponding substrates, causing the differences in the contact angles at different locations. The contact angles on Channels I and II surfaces are in the ranges of 166° to 173°, and 165° to 170°, respectively. The advanced and receding angles of the water drop were also measured by slightly pressing and lifting it, respectively, using the syringe. The maximum advanced angles and minimum receding angles measured at different locations of these two types of surfaces were 174° and 163°, respectively. Therefore, the maximum contact angle hysteresis is 11°. According to experimental results, there is no much difference between the two surfaces in their super-hydrophobic properties. This point indicates, although the larger spacing of the micropillars and the wider channels on the Channel II surface reduce the solid/water contact area, the Channel I surface has already had super-hydrophobic properties, as judged from its contact angles and contact angle hysteresis. Accordingly, the reduced solid/water contact area on the Channel II does not result in dramatic changes in super-hydrophobic properties. Similar results were also obtained in our previous work on different lotus surfaces [2].

**Figure 6:**
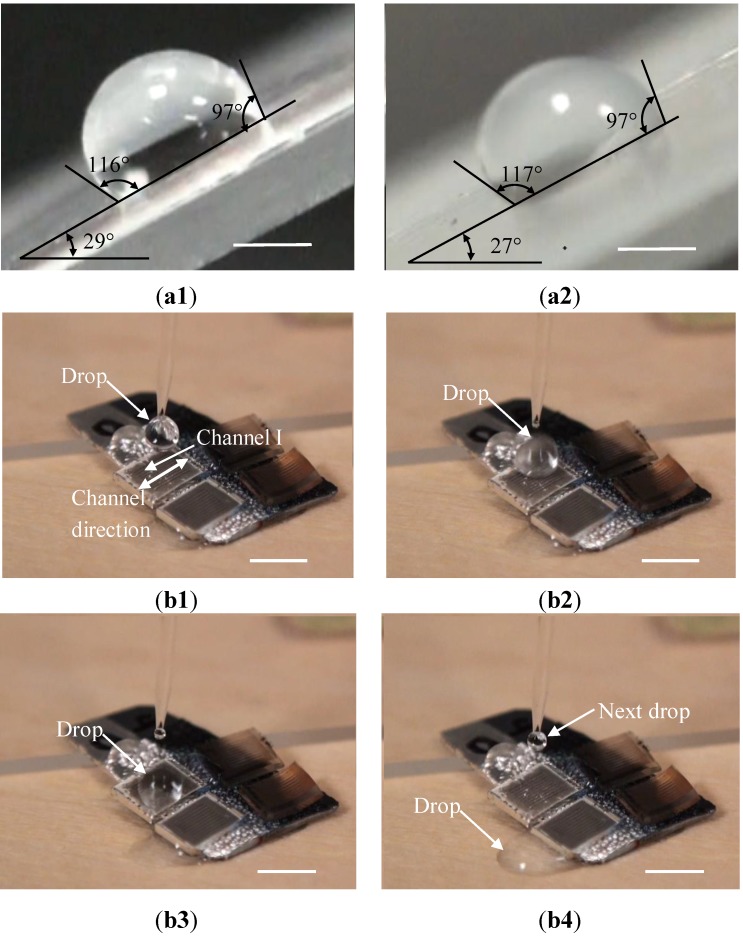
Water drops stuck to as-received PS sheets. (**a1**) Before; and (**a2**) After recovery when the sheets were tilted by 29° and 28°, respectively. The scale bars in (**a1**) and (**a2**) stand for 1 mm. A water drop rolled off from a substrate that is structured with Channels I when this substrate was tilted at an angle of 5°. (**b1**) Produce a water drop using a syringe, (**b2**) Release it to the substrate; (**b3**) The drop moves down; and (**b4**) Gets off from this substrate. The corresponding video clip for (**b1**–**b4**) is available in the Supporting Information, and the scale bars represent 4.5 mm.

We further examine whether water drops could easily roll off from a tilted surface. The minimum tilt angles for water drops to move down from as-received PS sheets before and after recovery were 30° to 29° respectively. As shown in Figure 6a, water drops still stuck to these sheets when the tilt angles were 29° to 28°, respectively. In contrast, we found that water drops quickly rolled off from Channels I and II substrates along the channel directions when the substrates were tilted by an angle of 1° or above. Furthermore, a water drop can also easily run across channels and get off from these two types of channel-structured substrates. For example, as shown in Figure 6b and in the corresponding video clip of the Supplemental Information, at a tilt angle of 5°, water drops quickly moved down from the Channel I substrate. The minimum tilt angle of a substrate that makes a water drop move down is related to contact angle hysteresis on the substrate, and increases with the increase in the contact angle hysteresis. Since the contact angle hysteresis for the channel-structured surfaces was about 10° less than those for the as-received PS sheets, the minimum tilt angles are reduced accordingly, implying that a water drop is easier to get off from the channel-structured surfaces, and that the corresponding wetting should be in Cassie-Baxter state.

In summary, the above testing results indicate that, due to the incorporation of hybrid surface structures, the generated channels, as well as the corresponding substrates, have super-hydrophobic properties.

## 4. Conclusions

A new approach was developed in this work to fabricate microchannels that are covered with hybrid micro/nanostructures. As in the case of a lotus leaf, these hybrid structures enable the corresponding microchannels to have super-hydrophobic properties. Such super-hydrophobic channels could be employed to reduce drag forces in microfluidic applications due to small solid/water contact areas between channel inner surfaces and water flows. In addition, the hybrid micro/nanostructures on the top surface of the substrate, together with these super-hydrophobic channels, can also be applied to enhance hydrophobicity of the substrate, making a water drop, for example, easily move down from this substrate, as demonstrated in the wetting tests.

## Supplementary Materials

Supplementary File 1

## References

[1] Neinhuis C., Barthlott W. (1997). Characterization and distribution of water-repellent, self-cleaning plant surfaces. Ann. Bot Lond..

[2] Xiang M.M., Wilhelm A., Luo C. (2013). Existence and role of large micropillars on the leaf surfaces of the president lotus. Langmuir.

[3] Wenzel R.N. (1936). Resistance of solid surfaces to wetting by water. Ind. Eng. Chem..

[4] Cassie A.B.D., Baxter S. (1944). Wettability of porous surfaces. Trans. Faraday Soc..

[5] Lauga E., Stone H.A. (2003). Effective slip in pressure-driven Stokes flow. J. Fluid Mech..

[6] Ou J., Perot B., Rothstein J.P. (2004). Laminar drag reduction in microchannels using ultrahydrophobic surfaces. Phys. Fluids.

[7] Ybert C., Barentin C., Cottin-Bizonne C., Joseph P., Bocquet L. (2007). Achieving large slip with superhydrophobic surfaces: Scaling laws for generic geometries. Phys. Fluids.

[8] Zhu L.B., Xiu Y.H., Xu J.W., Tamirisa P.A., Hess D.W., Wong C.P. (2005). Superhydrophobicity on two-tier rough surfaces fabricated by controlled growth of aligned carbon nanotube arrays coated with fluorocarbon. Langmuir.

[9] Koch K., Bhushan B., Jung Y.C., Barthlott W. (2009). Fabrication of artificial lotus leaves and significance of hierarchical structure for superhydrophobicity and low adhesion. Soft Matter.

[10] Ming W., Wu D., van Benthem R., de With G. (2005). Superhydrophobic films from raspberry-like particles. Nano Lett..

[11] Chong M.A.S., Zheng Y.B., Gao H., Tan L.K. (2006). Combinational template-assisted fabrication of hierarchically ordered nanowire arrays on substrates for device applications. Appl. Phys. Lett..

[12] Zhang Y., Sundararajan S. (2008). Superhydrophobic engineering surfaces with tunable air-trapping ability. J. Micromech. Microeng..

[13] Choi S.J., Suh K.Y., Lee H.H. (2008). A geometry controllable approach for the fabrication of biomimetic hierarchical structure and its superhydrophobicity with near-zero sliding angle. Nanotechnology.

[14] Jeong H.E., Kwak M.K., Park C.I., Suh K.Y. (2009). Wettability of nanoengineered dual-roughness surfaces fabricated by UV-assisted capillary force lithography. J. Colloid Interface Sci..

[15] Koch M., Evans A.G.R., Brunnschweiler A. (1999). Design and fabrication of a micromachined Coulter counter. J. Micromech. Microeng..

[16] Nellissen T., Wang L., Wehrens R., van den Heuvel E., Wererings J. A Novel Photolithographic Method for Realizing 3-D Interconnection Patterns on Electronic Modules. Proceedings of the 14th European Microelectronics and Packaging Conference.

[17] Pham N.P., Burghartz J.N., Sarro P.M. (2005). Spray coating of photoresist for pattern transfer on high topography surfaces. J. Micromech. Microeng..

[18] Su W., Lee S., Tsai M., Fang W. 3D Lithography and Deposition on Highly Structured Surfaces Using Plasma Surface Modification, SAM Coating, and Contact Displacement Electroless Plating. Proceedings of the 19th IEEE International Conference on Micro Electro Mechanical Systems.

[19] Wang H., Chakraborty A., Luo C. (2010). Fabrication of Au micropatterns on vertical Si sidewalls using flexible PDMS shadow masks. J. Micromech. Microeng..

[20] Wang H., Luo C. (2011). Generation of Au micropatterns on two sidewalls of a Si channel through a PDMS shadow mask. J. Micromech. Microeng..

[21] Luo C., Xiang M.M., Heng X. (2012). A stable intermediate wetting state after a water drop contacts the bottom of a microchannel or is placed on a single corner. Langmuir.

[22] Liu X.C., Luo C. (2009). Fabrication of Au sidewall micropatterns using Si-reinforced PDMS molds. Sens. Actuator A Phys..

[23] Liu X.C., Luo C. (2010). Fabrication of super-hydrophobic channels. J. Micromech. Microeng..

[24] Chakraborty A., Liu X.C., Luo C. (2012). Generation of sidewall patterns in microchannels via strain-recovery deformations of polystyrene. Sens. Actuator A Phys..

[25] Wang C.C., Huang W.M., Ding Z, Zhao Y., Purnawali H. (2012). Cooling-/water-responsive shape memory hybrids. Compos. Sci. Technol..

[26] Hidber P.C., Nealey P.F., Helbig W., Whitesides G.M. (1996). New strategy for controlling the size and shape of metallic features formed by electroless deposition of copper: Microcontact printing of catalysts on oriented polymers, followed by thermal shrinkage. Langmuir.

[27] Zhao X., Xia Y., Schueller O., Qin D., Whitesides G. (1998). Fabrication of microstructures using shrinkable PS films. Sens. Actuator A Phys..

[28] Grimes A., Breslauer D.N., Long M., Pegan J., Lee L.P., Khine M. (2008). Shrinky-Dink microfluidics: Rapid generation of deep and rounded patterns. Lab Chip.

[29] Liu X.C., Chakraborty A., Luo C. (2010). Fabrication of micropatterns on the sidewalls of a thermal shape memory polystyrene block. J. Micromech. Microeng..

[30] Luo C., Liu X.C., Poddar R., Garra J., Gadre A.P., Keuren E.V., Schneider T., White R., Currie J., Paranjape M. (2006). Thermal ablation of PMMA for water release using a microheater. J. Micromech. Microeng..

[31] Irie M., Otsuka K., Wayman C.M. (1998). Shape Memory Polymers. Shape Memory Materials.

[32] Bowden N., Brittain S., Evans A.G., Hutchinson J.W., Whitesides G.M. (1998). Spontaneous formation of ordered structures in thin films of metals supported on an elastomeric polymer. Nature.

[33] Zhao Y., Huang W.M., Fu Y.Q. (2011). Formation of micro/nano-scale wrinkling patterns atop shape memory polymers. J. Micromech. Microeng..

[34] Cheng M.C., Garra J.A., Gadre A.P., Nijdam A.J., Luo C., Paranjape M., Currie J.F., Schneider T., White R. (2004). Dry lease of polymer structures with anti-sticking layer. J. Vac. Sci. Technol. A.

